# Deciphering cork formation in *Quercus suber*

**DOI:** 10.1186/1753-6561-5-S7-P172

**Published:** 2011-09-13

**Authors:** Jorge AP Paiva, Pedro Fevereiro, Pedro Marques, José Carlos Rodrigues, Grégoire Le Provost, Christophe Plomion, Jacqueline Grima-Pettenati, Olivier Bouchez, Christophe Klopp, Hélène Berges, José Graça

**Affiliations:** 1 Instituto de Investigação Científica Tropical (IICT), FLOR-Centro de Florestas e dos Produtos Florestais, Tapada da Ajuda, 1349-018 Lisboa, Portugal & Instituto de Biologia Experimental e Tecnológica, Apartado 12, 2781-901 Oeiras, Portugal; 2 Instituto de Biologia Experimental e Tecnológica, Apartado 12, 2781-901 Oeiras, Portugal; 3 Fundação João Lopes Fernandes (FJLF), Herdade do Leitões 7425-014 Montargil, Portugal; 4 Instituto de Investigação Científica Tropical (IICT), FLOR-Centro de Florestas e dos Produtos Florestais, Tapada da Ajuda, 1349-018 Lisboa, Portugal; 5 INRA-UMR 1202 - BIOGECO - "BIOdiversité, Gènes et Communautés" (INRA-UMR BIOGECO) Site de Recherches Forêt Bois de Pierroton, 69 route d'Arcachon, 33612CESTAS Cedex, France; 6 UMR CNRS/Université Toulouse III 5546, Pôle de Biotechnologies Végétales, 24 chemin de Borde Rouge, BP42617 Auzeville, 31326 Castanet Tolosan, France; 7 Plateforme Génomique, Génopole Toulouse/Midi-pyrénées, INRA Auzeville, Chemin de Borderouge-BP 52627, 31326 Castanet-Tolosan, France; 8 INRA-Toulouse, BIA, 31326 Castanet-Tolosan, France; 9 INRA-CNRGV, Chemin de Borde Rouge, 31326 Castanet-Tolosan, France; 10 Instituto Superior de Agronomia (ISA/UTL) Tapada da Ajuda 1349-017Lisboa, Portugal

## 

The family of *Fagaceae*, comprises about 900 species, among them the best-known group of this family is the oaks, genus *Quercus*, that are commonly used as timber or for cork production. Cork is produced by *Q. suber*, an evergreen oak with major economic and environmental importance for Mediterranean region, in particular for Portugal that is the leading producer of this material.

Besides, the economic importance of cork production and cork manufacturing, little attention has been paid to the molecular mechanisms underlying wood and cork formation as well as cork quality. To overcome this constraint, an international partnership (SuberGene) was established 2008, involving Portuguese and French research institutions and one Producer’s Association organization (FJLF). The main driving force of this partnership is to join efforts and complementary skills to unravel to the molecular mechanisms underlying cork formation and decipher the structural polymorphisms and regulation network that determine cork quality.

A non-normalized cDNA library of developing phellem (DP) was produced and 5,000 clones were sequence both ends using Sanger technology. More than 6500 good quality ESTs were deposit at GeneBank. DP transcriptome was also assessed by pirosequencing (454FLX Titanium, Roche) generating more than 200,000 reads. Sequencing data (238,911 ESTs) data were assembled into 69,559 contigs. Suberin biosynthesis genes such as Glycerol-3-phosphate acyltransferase and Omega-hydroxypalmitate O-feruloyl transferase are among the more expressed genes in DP tissues. More than 2,800 putative SNPs were detected in 1,121 contigs. As a complementary strategy, the proteome of DP are being assessed, by 2D-PAGE, and mass spectroscopy. In order to get more information about the gene structure of genes related with the suberisation of cork cell-wall, one *Q. suber* BAC library have been constructed, and are being characterized. Clones harboring genes of interest were identified by screening high-density filters of this BAC library.

These resources provides us with valuable tools to study the nature of the molecular machinery involved in cork formation,and most importantly with the players involved in the variability of cork characteristics. Future ongoing approaches and the impact of these finding will be discussed.

